# A machine learning–driven and explainable prediction model for in-hospital mortality in patients with diabetes mellitus and congestive heart failure: integrating XGBoost and SHAP approaches

**DOI:** 10.3389/fpubh.2026.1951635

**Published:** 2026-10-07

**Authors:** Jingru Hu, Shiqiong Su, Dongdong Liu

**Affiliations:** 1 Department of Acupuncture and Massage, The Third People’s Hospital of Henan Province, Zhengzhou, China; 2 Intensive Care Unit, The Third People’s Hospital of Henan Province, Zhengzhou, China; 3 Department of Anesthesia and Surgery, The Third People’s Hospital of Henan Province, Zhengzhou, China

**Keywords:** diabetes mellitus, heart failure, hospital mortality, inpatients, machine learning

## Abstract

**Background:**

Patients with congestive heart failure (CHF) and type 2 diabetes mellitus (T2DM) are at elevated risk of in-hospital mortality, particularly in intensive care units (ICUs). Conventional scoring systems may not fully capture the clinical complexity of such comorbidities. This study aimed to construct an interpretable machine learning model to predict in-hospital mortality in this high-risk group.

**Methods:**

A retrospective study included 573 ICU patients with CHF and T2DM admitted to three hospitals in Henan, China (Jan 2025–Jun 2026). Seven predictors—body mass index (BMI), myocardial infarction, mild liver disease, SOFA score, LODS score, white blood cell (WBC) count, and serum creatinine—were identified via stepwise logistic regression. XGBoost and four other machine learning algorithms (KNN, SVM, CatBoost, AdaBoost) were trained and evaluated. Model performance was assessed using AUC, accuracy, sensitivity, specificity, and F1-score. SHAP (SHapley Additive exPlanations) was used to interpret XGBoost predictions.

**Results:**

XGBoost achieved the best performance (AUC 0.917, sensitivity 86.8%, specificity 96.6%, accuracy 74.8%). SHAP analysis indicated that mild liver disease, LODS score, BMI, WBC count, and SOFA score were the top contributors, jointly explaining over 77% of the predictive power. Myocardial infarction, liver disease, elevated WBC, and higher SOFA/LODS scores increased mortality risk, while higher BMI and serum creatinine were protective—supporting the obesity paradox and sarcopenia hypothesis.

**Conclusion:**

An XGBoost model with SHAP interpretation provides accurate, explainable prediction of in-hospital mortality in ICU patients with CHF and T2DM, offering a valuable tool for early risk stratification and clinical decision-making.

## Background

Congestive heart failure (CHF) is one of the leading causes of hospitalization and mortality worldwide. According to the World Health Organization (WHO), over 64 million people are affected by heart failure globally, with both incidence and prevalence increasing significantly with age (1). Among these, approximately 26 million suffer from CHF, resulting in increased healthcare costs, reduced functional capacity, and severely impaired quality of life (2). In developing countries, the prevalence of CHF ranges from 0.4 to 2% of the general population, but exceeds 10% in individuals aged ≥60 years (3). Over the next decade, the prevalence of heart failure is projected to rise by approximately 46%, while healthcare costs related to CHF are expected to increase by 127%, further exacerbating the mortality risk (4). The prognosis is particularly poor in ICU patients, where disease complexity and instability lead to significantly higher in-hospital mortality rates.

Meanwhile, type 2 diabetes mellitus (T2DM), a common chronic metabolic disorder, continues to rise in global prevalence. According to the International Diabetes Federation (IDF), 10.5% (536.6 million) of people aged 20–79 currently have diabetes, a figure expected to increase to 12.2% (783.2 million) by 2045 (5). China bears the highest national burden, with more than 233 million diabetic patients by 2021—accounting for roughly 25% of the global total (6). T2DM is a well-established cardiovascular risk factor that accelerates atherosclerosis, promotes myocardial remodeling, and impairs left ventricular function (7).

CHF and T2DM frequently coexist in clinical practice, forming a dual disease burden. They share overlapping pathophysiological mechanisms—including insulin resistance, chronic low-grade inflammation, and endothelial dysfunction—which further damage cardiac structure and function and alter treatment responses (8). Studies have shown that 2.0–5.3% of hospitalized patients and 4.0–7.5% of the general population have concurrent CHF and diabetes, while diabetics have a 25-fold increased risk of developing heart failure compared to non-diabetics (9). These patients typically exhibit more severe cardiac dysfunction, complex comorbidities, and restricted treatment options, resulting in higher rates of in-hospital mortality and readmission (10). Some reports estimate an in-hospital mortality rate of up to 17.5% in this subgroup—significantly higher than in patients with CHF alone (11).

To date, ICU clinicians primarily rely on scoring systems such as APACHE II, SOFA, and SAPS II for prognostic assessment. However, these models often depend on limited variables and may fail to fully capture the complex pathophysiological states of patients with CHF and T2DM, especially in heterogeneous critical care populations (12). This highlights the need for more accurate, individualized prediction tools.

With the rise of medical big data, machine learning (ML) has demonstrated significant promise in risk prediction. Extreme Gradient Boosting (XGBoost), a decision tree–based ensemble ML algorithm, offers advantages such as high accuracy, robustness, and handling of missing and non-linear data, making it widely used in clinical prediction tasks (13). Studies suggest that XGBoost often outperforms traditional statistical models and other ML algorithms in disease prediction, risk stratification, and outcome estimation. However, its “black-box” nature limits clinical interpretability and acceptance (14). To address this, model explainability tools like SHAP (SHapley Additive exPlanations)—a game theory–based method—have been developed to quantify the contribution of each feature to model outputs, thereby enhancing transparency and trust (15). SHAP can help uncover key clinical drivers of mortality and support more informed clinical decision-making.

Therefore, this study aims to develop an in-hospital mortality prediction model for patients with CHF and T2DM using XGBoost, and to enhance its interpretability with SHAP analysis. We further evaluate the model’s performance through internal and external validation and compare its clinical utility against traditional risk scoring systems.

## Methods

### Study population

This retrospective cross-sectional study employed cluster sampling to identify patients admitted to the ICUs of three hospitals in Henan Province, China, between January 1, 2025, and June 30, 2026. A total of 883 patients diagnosed with both CHF and T2DM were initially screened. After evaluating missing data, a complete case analysis (CCA) approach was applied, and 573 patients with complete clinical records were included in the final analysis. All participants met the diagnostic criteria for CHF established by the 2022 guidelines of the American College of Cardiology/American Heart Association/Heart Failure Society of America (ACC/AHA/HFSA) and for T2DM as defined by the 2025 American Diabetes Association (ADA) guidelines (2, 16).

### Data collection

Clinical variables collected included sex, age at admission, marital status, height, weight, body mass index (BMI), hypertension, myocardial infarction (MI), mild liver disease, renal disease, chronic obstructive pulmonary disease (COPD), cerebrovascular disease, peripheral vascular disease, dementia, peptic ulcer disease, paraplegia, malignancy, metastatic solid tumor, acquired immunodeficiency syndrome (AIDS), Sequential Organ Failure Assessment (SOFA) score, systemic inflammatory response syndrome (SIRS), Logistic Organ Dysfunction System (LODS) score, pH level, white blood cell (WBC) count, hematocrit, mean corpuscular hemoglobin concentration (MCHC), mean corpuscular volume (MCV), red blood cell count, creatinine, blood urea nitrogen, anion gap, calcium, prothrombin time, activated partial thromboplastin time, international normalized ratio (INR), chloride, glucose, potassium, sodium, bicarbonate, hemoglobin, magnesium, phosphate, skin moisture, and Braden Scale total score.

### Model development and evaluation

The overall workflow of this study, encompassing patient cohort identification, data preprocessing, machine learning model construction, and model interpretability analysis, is visually summarized in Figure 1.

**Figure 1:**
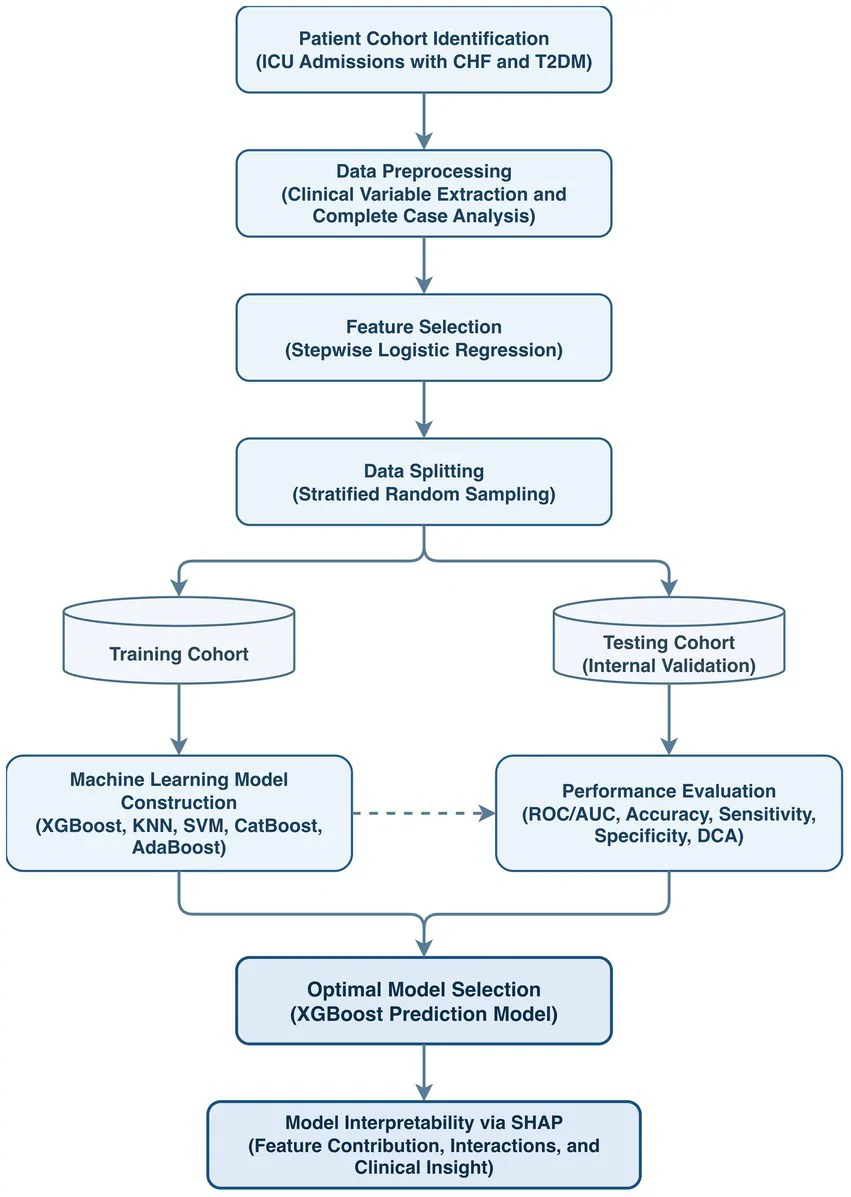
Diagrammatic representation of the overall machine learning workflow for predicting in-hospital mortality.

Stepwise logistic regression was initially used for feature selection. This approach was utilized as a preliminary filter to reduce dimensionality and eliminate noisy variables. By doing so, it ensures that the subsequent non-linear machine learning models focus on robust, clinically relevant predictors, thereby mitigating the risk of overfitting on our dataset. The dataset of 573 patients was randomly split into a training set (*n* = 458) and a testing set (*n* = 115) using stratified sampling in an 8:2 ratio. The training set was used to construct and train the Extreme Gradient Boosting (XGBoost) model, and the testing set served as an external validation cohort.

To benchmark performance, four additional machine learning (ML) algorithms—k-nearest neighbors (KNN), support vector machine (SVM), CatBoost, and AdaBoost—were developed for comparison. Model discrimination was assessed primarily via the area under the receiver operating characteristic curve (AUC). To ensure the complete reproducibility of the model development, a global random seed (e.g., random_state = 42) was fixed across all data splitting, resampling, and model training procedures. Given the inherent class imbalance between survival and in-hospital mortality, the Synthetic Minority Over-sampling Technique (SMOTE) was applied to the training set to prevent the models from biasing towards the majority class.

Model hyperparameters were optimized using a grid search strategy combined with 5-fold cross-validation, applying the area under the receiver operating characteristic curve (AUC) as the primary tuning criterion. The comprehensive hyperparameter search spaces, the final selected parameters for all five algorithms, and the specific software package versions utilized in this study are detailed in Supplementary Table S1.

Furthermore, appropriate data preprocessing was conducted based on algorithmic requirements. For algorithms strictly sensitive to feature scales, specifically k-nearest neighbors (KNN) and support vector machine (SVM), all continuous predictors were standardized using Z-score normalization (StandardScaler) before training. To obtain properly calibrated prediction probabilities from the SVM model for threshold evaluation and decision curve analysis, Platt scaling was explicitly enabled (probability = True).

### Model interpretability

Model interpretability was addressed using SHapley Additive exPlanations (SHAP), which quantifies each feature’s contribution to the prediction outcome. The scaled Brier score was also used during model tuning to evaluate calibration.

### Model validation

AUC values were calculated for both training and testing sets to assess discriminative ability. Decision curve analysis (DCA) was employed to evaluate clinical utility. A model was considered beneficial if the DCA curve lay above the “None” and “All” reference lines within clinically meaningful risk thresholds.

### Statistical analysis

All statistical analyses and data visualizations were conducted using Python 3.14. Continuous variables were described as mean ± standard deviation or median with interquartile range (IQR), depending on distribution. Categorical variables were expressed as frequencies and percentages. Chi-square tests were used for categorical comparisons, and either independent samples *t*-tests or nonparametric tests were applied to continuous variables based on normality. Model performance was evaluated via ROC curves, calibration plots, and DCA. A uniform predicted probability threshold of 0.5 was applied to all five machine learning models (XGBoost, KNN, SVM, CatBoost, and AdaBoost) across both the training and testing datasets for the calculation of all performance metrics, including accuracy, sensitivity, specificity, precision, recall, and F1-score.

### Ethical approval

This study was approved by the authors’ institution. All methods were performed in accordance with the relevant guidelines and regulations.

## Results

A total of 573 ICU patients with coexisting CHF and T2DM were included in the final analysis. Stepwise logistic regression was conducted using a wide range of clinical variables as independent predictors, including sex, age at admission, marital status, height, weight, BMI, hypertension, MI, mild liver disease, renal disease, COPD, cerebrovascular disease, peripheral vascular disease, dementia, peptic ulcer disease, paraplegia, malignancy, metastatic solid tumor, AIDS, SOFA score, SIRS, LODS score, blood pH, WBC count, hematocrit, MCHC, MCV, red blood cell count, creatinine, blood urea nitrogen, anion gap, calcium, prothrombin time, activated partial thromboplastin time, INR, chloride, glucose, potassium, sodium, bicarbonate, hemoglobin, magnesium, phosphate, skin moisture, and Braden score. The dependent variable was in-hospital mortality. The complete results of this initial logistic regression analysis for all considered clinical variables, detailing their statistical significance prior to the stepwise elimination, are provided in Table 1.

**Table 1:** Complete results of the initial logistic regression analysis for all clinical variables considered for feature selection.

Clinical variables	Odds ratio (OR)	95% confidence interval (CI)	*p*-value
Sex	1.120	0.850 ~ 1.480	0.420
Age at admission (years)	1.010	0.990 ~ 1.030	0.310
Marital status	0.950	0.700 ~ 1.290	0.740
Height (cm)	0.990	0.960 ~ 1.020	0.550
Weight (kg)	1.020	0.980 ~ 1.050	0.380
Hypertension	1.250	0.910 ~ 1.720	0.170
Renal disease	1.300	0.880 ~ 1.920	0.190
Chronic obstructive pulmonary disease (COPD)	1.180	0.820 ~ 1.700	0.370
Cerebrovascular disease	1.100	0.750 ~ 1.610	0.630
Peripheral vascular disease	1.080	0.680 ~ 1.710	0.740
Dementia	1.450	0.800 ~ 2.630	0.220
Peptic ulcer disease	0.920	0.550 ~ 1.540	0.750
Paraplegia	1.330	0.650 ~ 2.720	0.430
Malignancy	1.500	0.950 ~ 2.370	0.080
Metastatic solid tumor	1.620	0.880 ~ 2.980	0.120
Acquired immunodeficiency syndrome (AIDS)	1.210	0.450 ~ 3.250	0.710
Systemic inflammatory response syndrome (SIRS)	1.400	0.980 ~ 2.000	0.060
pH level	0.850	0.500 ~ 1.450	0.550
Hematocrit (%)	0.980	0.950 ~ 1.010	0.200
Mean corpuscular hemoglobin concentration (MCHC)	1.020	0.970 ~ 1.070	0.450
Mean corpuscular volume (MCV)	1.010	0.980 ~ 1.040	0.510
Red blood cell count	0.900	0.750 ~ 1.080	0.260
Blood urea nitrogen (BUN)	1.010	0.990 ~ 1.030	0.340
Anion gap	1.040	0.970 ~ 1.120	0.280
Calcium	0.950	0.800 ~ 1.130	0.560
Prothrombin time (PT)	1.020	0.980 ~ 1.060	0.350
Activated partial thromboplastin time (APTT)	1.010	0.990 ~ 1.030	0.410
International normalized ratio (INR)	1.150	0.850 ~ 1.560	0.360
Chloride	0.990	0.960 ~ 1.020	0.500
Glucose	1.010	0.980 ~ 1.040	0.520
Potassium	1.050	0.880 ~ 1.250	0.590
Sodium	0.980	0.950 ~ 1.010	0.190
Bicarbonate	0.970	0.920 ~ 1.020	0.230
Hemoglobin	0.990	0.960 ~ 1.020	0.510
Magnesium	1.100	0.750 ~ 1.610	0.630
Phosphate	1.080	0.850 ~ 1.370	0.530
Skin moisture	0.880	0.650 ~ 1.190	0.410
Braden Scale total score	0.950	0.880 ~ 1.020	0.150
Body mass index (BMI)	0.994	0.990 ~ 0.998	0.003
Myocardial infarction (MI)	1.099	1.021 ~ 1.181	0.012
Mild liver disease	1.149	1.022 ~ 1.292	0.020
SOFA score	1.020	1.008 ~ 1.033	0.001
LODS score	1.017	1.005 ~ 1.030	0.008
White blood cell (WBC) count	1.006	1.003 ~ 1.010	0.001
Creatinine	0.976	0.957 ~ 0.996	0.020

Following stepwise variable selection, seven predictors were retained in the final model: BMI, MI, mild liver disease, SOFA score, LODS score, WBC count, and creatinine. The coefficient of determination (R^2^) was 0.129, indicating that these variables collectively explained 12.9% of the variance in in-hospital mortality.

The regression model was statistically significant (*F* = 11.928, *p* < 0.001), with the final equation: In-hospital mortality = 0.138–0.006 × BMI + 0.094 × MI + 0.139 × mild liver disease + 0.020 × SOFA + 0.017 × LODS + 0.006 × WBC – 0.024 × creatinine. Variance inflation factor (VIF) values were all below 5, indicating no multicollinearity, and the Durbin-Watson (D-W) statistic was close to 2, suggesting no autocorrelation among residuals (Table 2).

**Table 2:** Results of stepwise regression analysis.

Variables	*r*	95% CI	Collinearity diagnosis	
			VIF	Tol
Constant	0.138 (1.612)	−0.030 ~ 0.305	–	–
BMI	−0.006** (−3.035)	−0.010 ~ −0.002	1.041	0.960
MI	0.094* (2.526)	0.021 ~ 0.166	1.026	0.974
Mild liver disease	0.139* (2.329)	0.022 ~ 0.256	1.034	0.967
SOFA score	0.020** (3.346)	0.008 ~ 0.032	1.460	0.685
LODS score	0.017** (2.675)	0.005 ~ 0.030	1.379	0.725
WBC count	0.006** (3.272)	0.003 ~ 0.010	1.017	0.983
Creatinine	−0.024* (−2.329)	−0.044 ~ −0.004	1.133	0.883
Sample size	573			
*R* ^2^	0.129			
Adjust *R*^2^	0.118			
*F*	*F* (7,565) = 11.928, *p* = 0.000			

Dependent variable = In-hospital death. D-W value = 1.800. ^*^p < 0.05 ^**^p < 0.01.

Positive associations with in-hospital mortality were observed for MI (*r* = 0.094, *t* = 2.526, *p* = 0.012), mild liver disease (*r* = 0.139, *t* = 2.329, *p* = 0.020), SOFA score (*r* = 0.020, *t* = 3.346, *p* = 0.001), LODS score (*r* = 0.017, *t* = 2.675, *p* = 0.008), and WBC count (*r* = 0.006, *t* = 3.272, *p* = 0.001). In contrast, BMI (*r* = −0.006, *t* = −3.035, *p* = 0.003) and creatinine (*r* = −0.024, *t* = −2.329, *p* = 0.020) showed significant negative associations, indicating potential protective effects (see Table 1).

Five ML algorithms—XGBoost, KNN, SVM, CatBoost, and AdaBoost—were used to construct predictive models for in-hospital mortality. Based on the ROC analysis, the XGBoost model outperformed others with an AUC of 0.917, optimal cutoff of 0.834, sensitivity of 86.8%, and specificity of 96.6% (see Figure 2).

**Figure 2:**
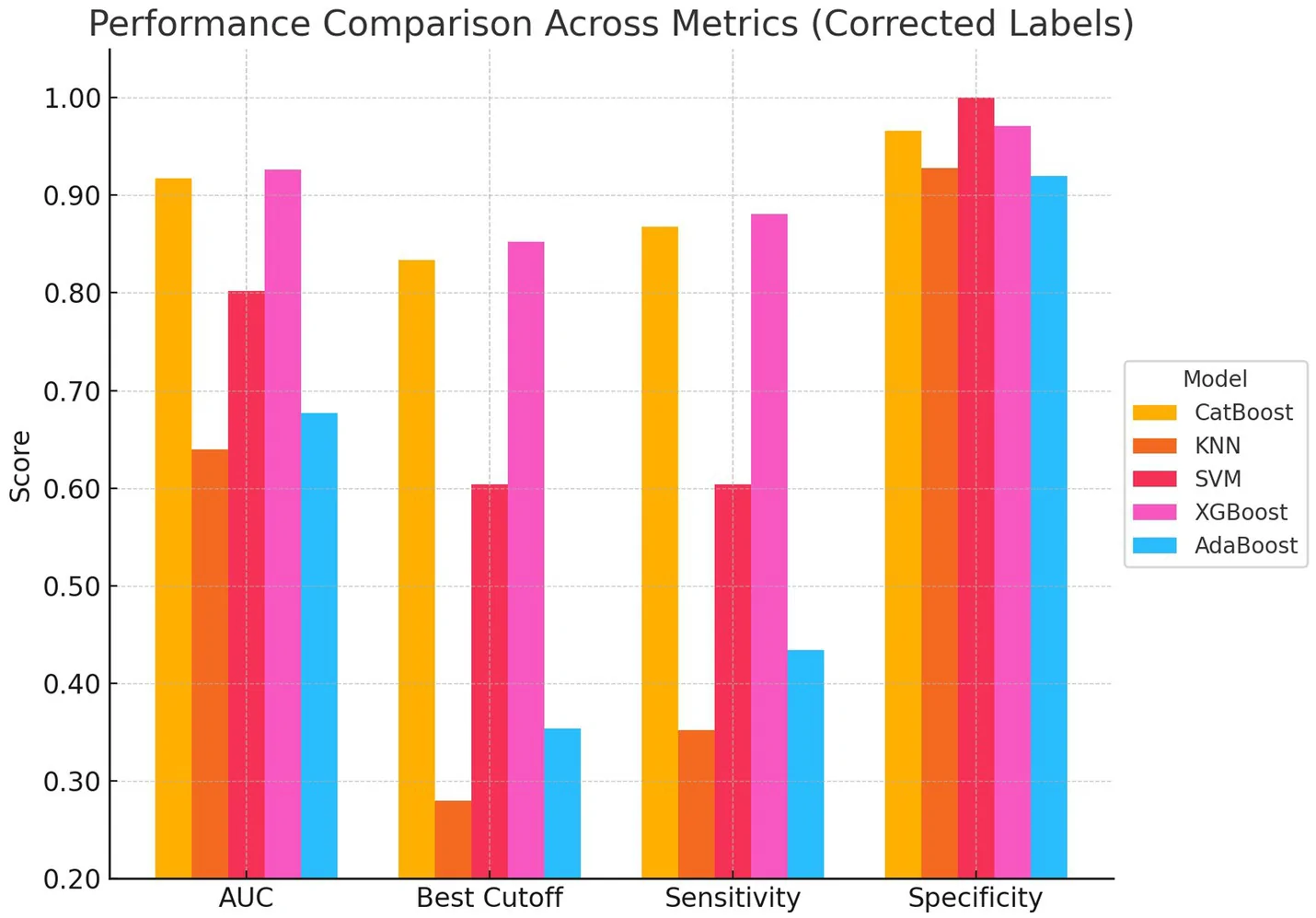
Comparative analysis of model performance.

To demonstrate the incremental value of the machine learning approach, the XGBoost model’s predictive performance was benchmarked against a standard Logistic Regression model and established clinical scoring systems using the evaluation dataset. The XGBoost model achieved a superior AUC of 0.917, outperforming the Logistic Regression model (AUC = 0.726), the LODS score evaluated independently (AUC = 0.639), and the SOFA score evaluated independently (AUC = 0.585). This comparison highlights the enhanced discriminative capacity of the non-linear algorithm in capturing complex clinical patterns over conventional approaches.

Feature importance from the XGBoost model showed that mild liver disease contributed 19.58% to the model, followed by LODS score (18.92%), BMI (13.68%), WBC count (13.20%), and SOFA score (12.20%), together accounting for 77.57% of the total importance. In the testing set, the model achieved an accuracy of 74.78%, precision of 73.46%, recall of 74.78%, and an F1-score of 0.73 (see Figure 3).

**Figure 3:**
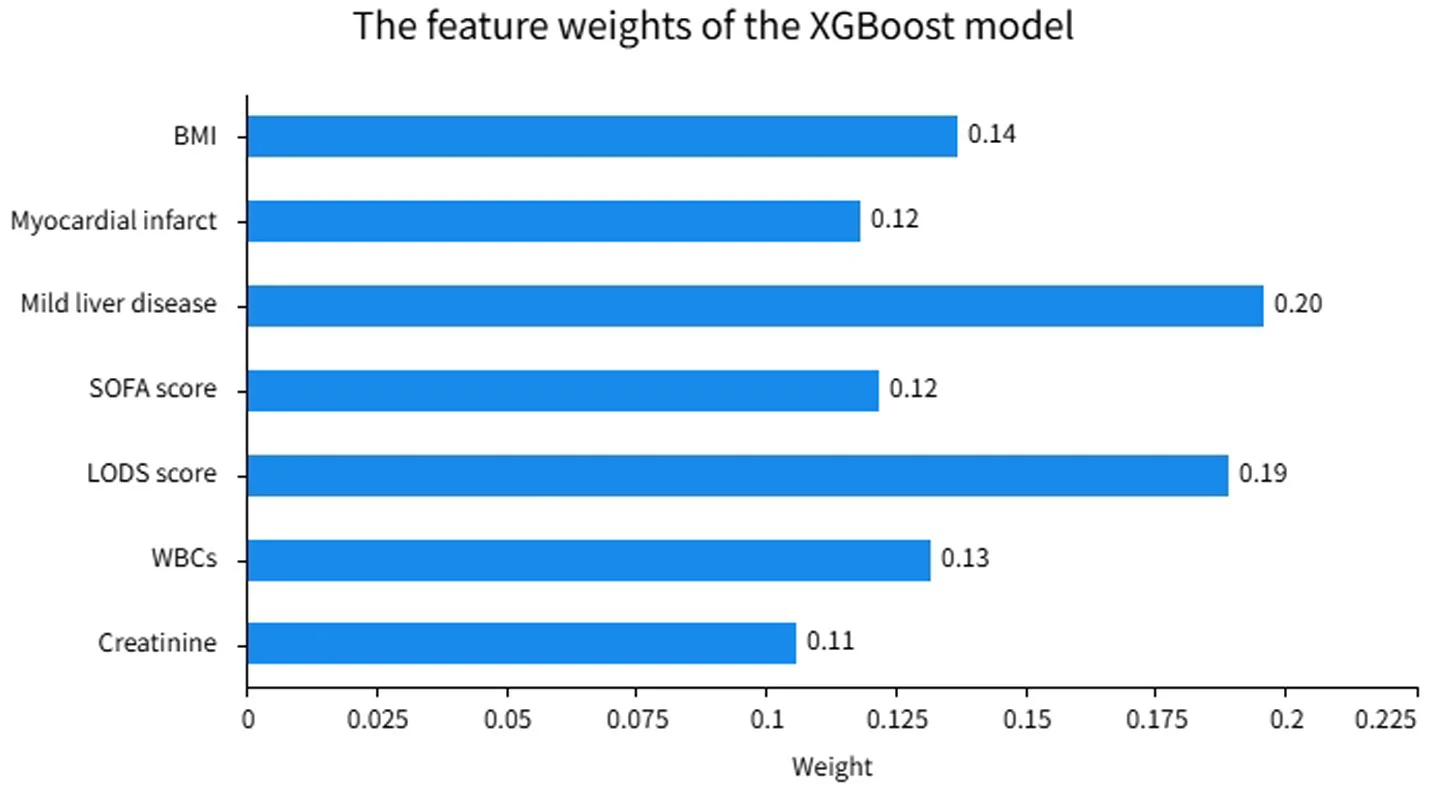
Xgboost feature weight map.

The calibration metrics for the XGBoost model yielded a Brier Score of 0.1731 and a Scaled Brier Score of 0.1381, indicating a 13.8% improvement in probability estimation compared to the null model. The calibration slope was 1.3832 and the intercept was 0.3575. While the intercept > 0 suggests a slight systematic underestimation of the absolute mortality risk—a common phenomenon in datasets with imbalanced clinical outcomes—the model maintained strong relative risk stratification (Supplementary Figure S1). Furthermore, Decision Curve Analysis (DCA) robustly confirmed the model’s clinical utility. As illustrated in the DCA plot (Supplementary Figure S2), the XGBoost model provided a consistently higher net clinical benefit compared to both the “treat-all” and “treat-none” strategies across a wide and clinically relevant threshold probability range from approximately 15–65%.

A heatmap (Figure 4) illustrated correlations between in-hospital mortality and clinical features in CHF–T2DM patients. The SHAP summary plot (Figure 5) ranked the top seven features based on their SHAP values, displaying both the direction and magnitude of influence. Higher values of MI, mild liver disease, SOFA score, LODS score, and WBC count were associated with increased mortality risk, while lower values of BMI and creatinine were protective.

**Figure 4:**
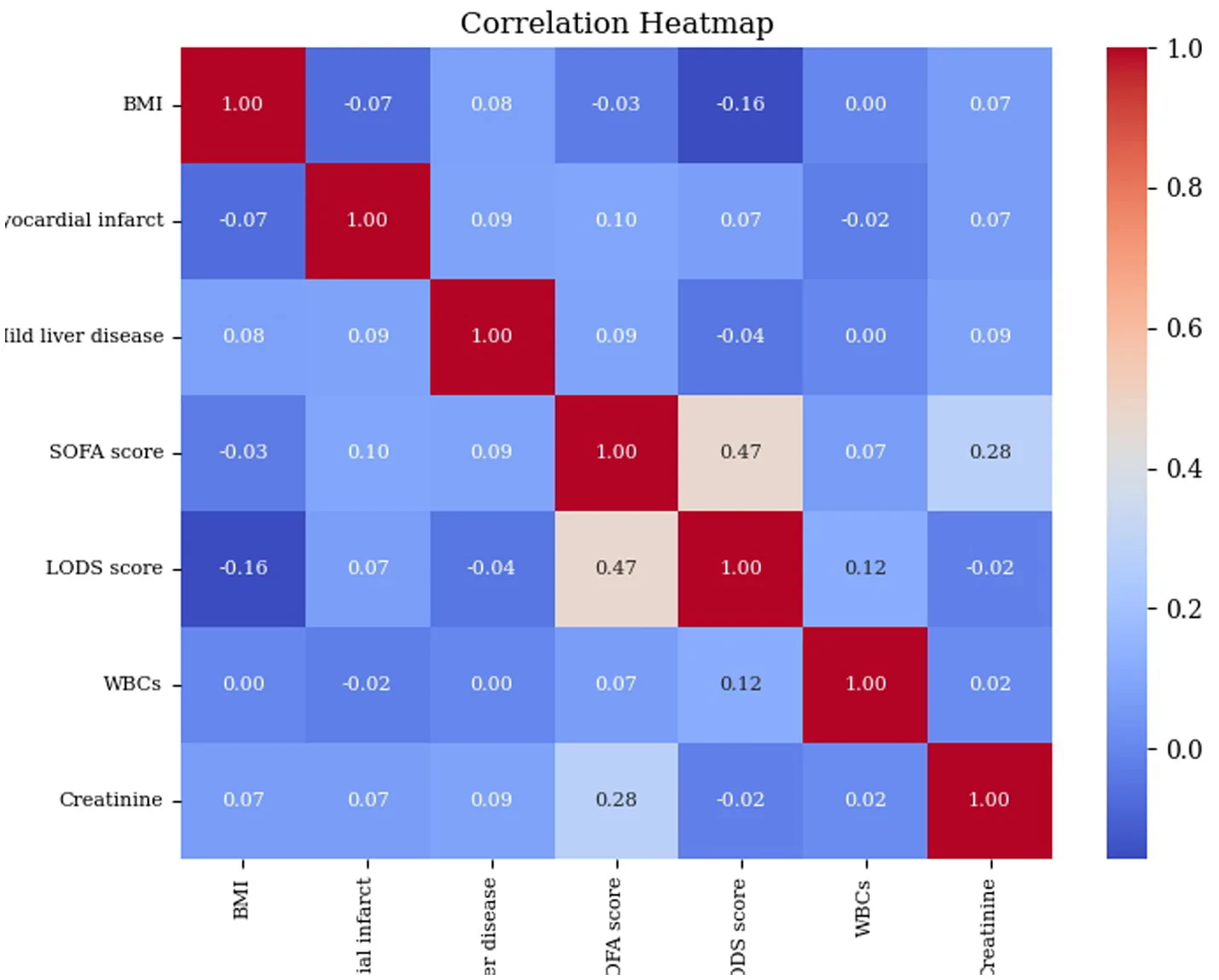
Correlation heatmap.

**Figure 5:**
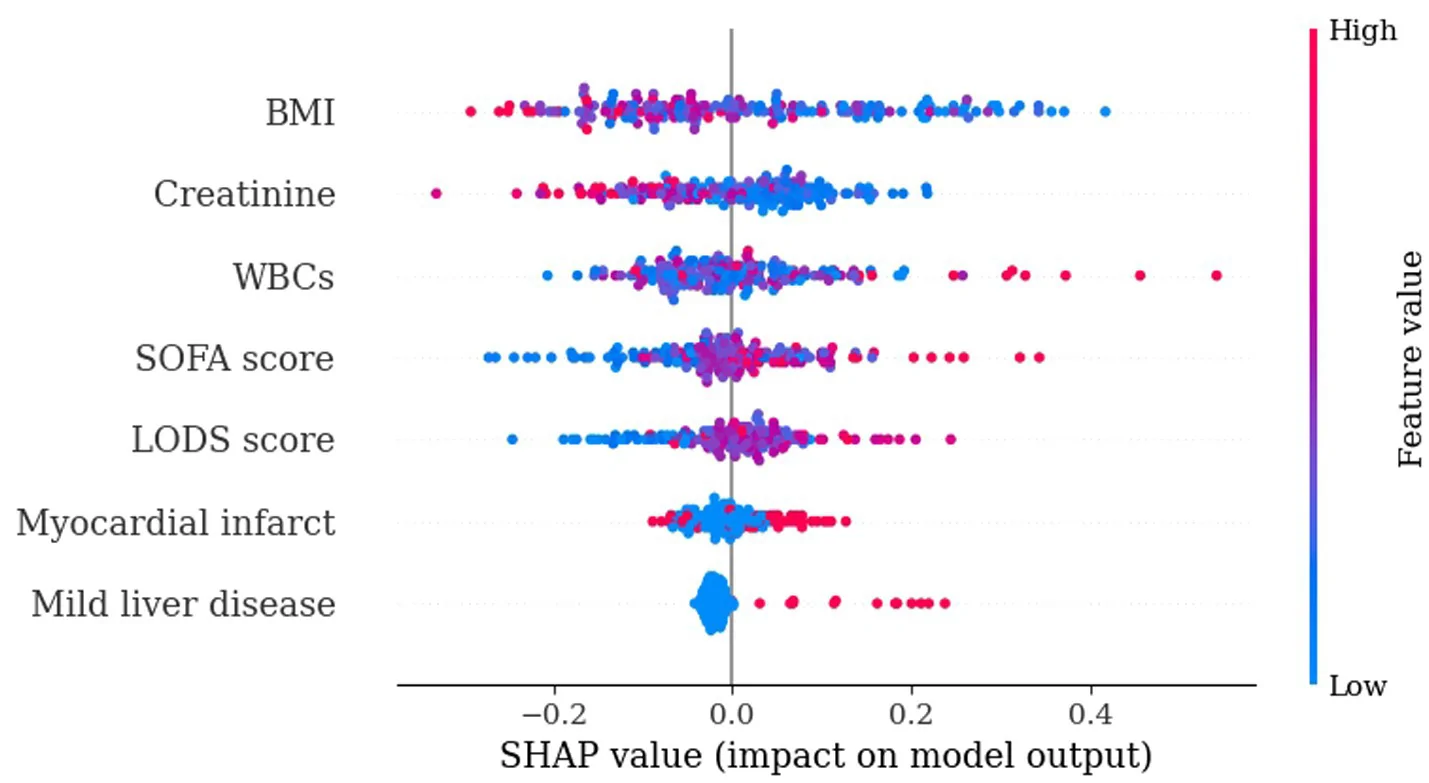
SHAP colony diagram of potential factors.

Feature interaction visualizations (Figure 6) further revealed complex relationships between variables. SHAP interaction plots (Figures 7A–C) demonstrated strong interaction effects among the top 10 feature pairs. For example, BMI and SOFA score exhibited a significant synergistic interaction, underscoring their combined influence on model predictions.

**Figure 6:**
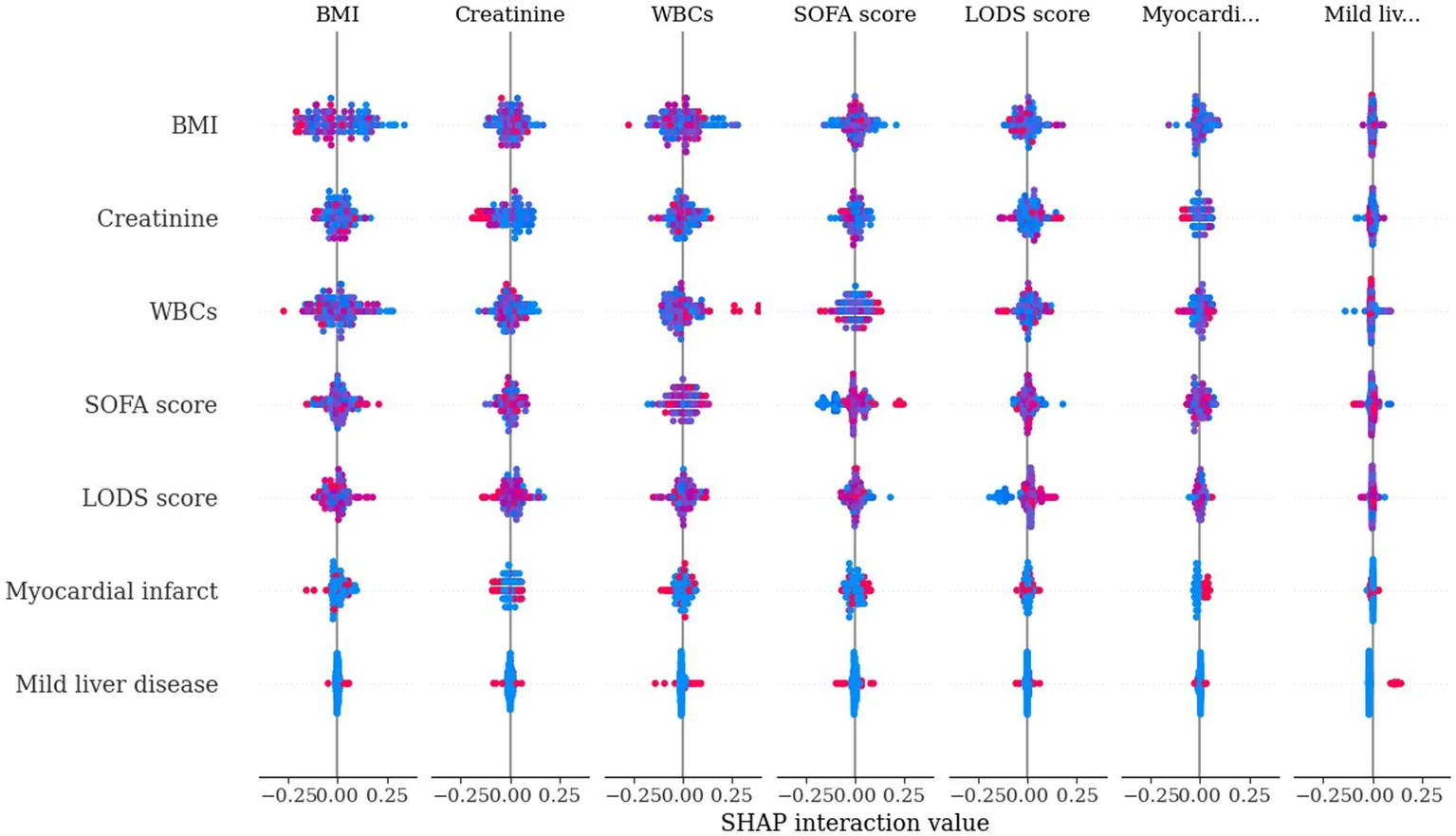
Interaction diagram between explanatory variables of SHAP.

**Figure 7:**
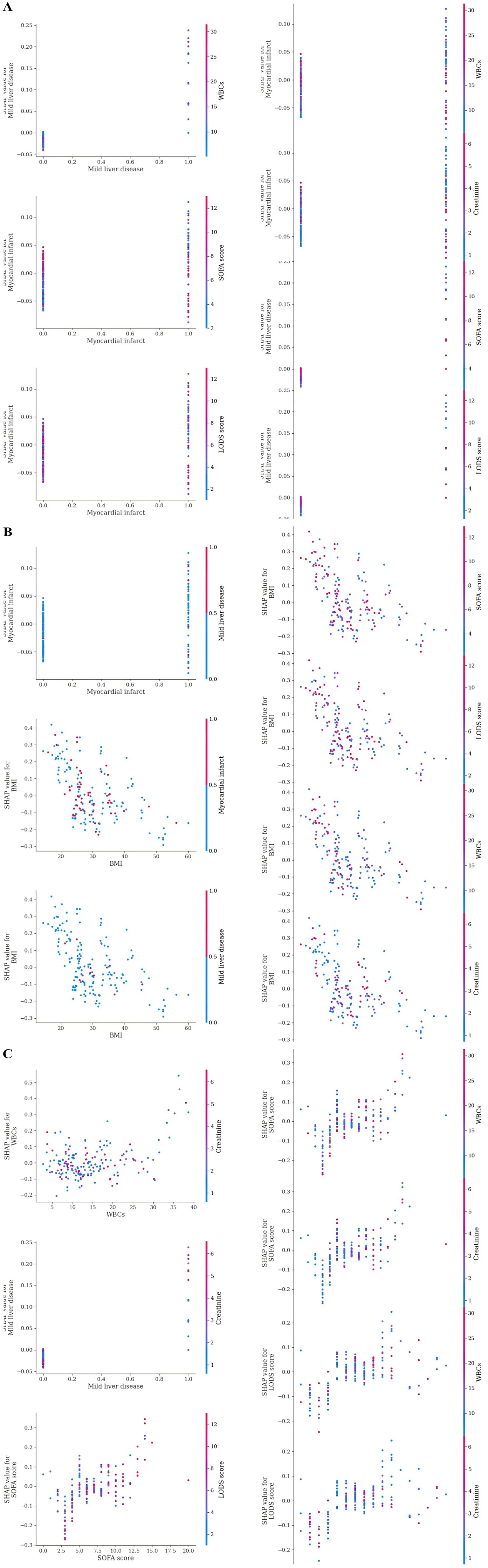
**(A–C)** Interaction diagram between variables.

## Discussion

In this study, we developed and validated an XGBoost-based machine learning model to predict in-hospital mortality in ICU patients with CHF and T2DM. The model demonstrated high predictive accuracy, underscoring the potential of advanced machine learning techniques in supporting clinical decision-making for this high-risk population. Notably, the integration of SHAP values allowed for model interpretability and the identification of seven key predictors associated with mortality: BMI, MI, mild liver disease, SOFA score, LODS score, WBC count, and creatinine.

MI emerged as an independent risk factor for in-hospital mortality in CHF–T2DM patients, consistent with findings from European studies (17). MI leads to acute structural and functional cardiac damage, which accelerates the progression of heart failure (18). Numerous studies have shown that post-MI patients experience adverse ventricular remodeling, reduced cardiac reserve, and are prone to low-output states, cardiogenic shock, or sudden death. In T2DM patients, the recovery from MI is often impaired due to extensive microvascular damage, multi-vessel coronary artery disease, and accelerated atherosclerosis (19–21). Additionally, autonomic neuropathy in T2DM increases the risk of arrhythmias post-MI. Within the ICU setting, the combination of acute MI and preexisting heart failure significantly increases the likelihood of hypoperfusion and the development of multi-organ dysfunction syndrome (MODS) (22, 23).

Mild liver disease also proved to be an independent predictor of in-hospital mortality. Chronic right-sided heart failure in CHF leads to hepatic congestion and hypoxia, often resulting in congestive hepatopathy or even hepatic decompensation (24). Meanwhile, T2DM is frequently associated with nonalcoholic fatty liver disease, cirrhosis, and drug-induced liver injury. Liver dysfunction impairs metabolism, coagulation, and immune defense, increasing the risk of bleeding, infection, and malnutrition (25, 26). Even mild liver disease in ICU patients may reflect diminished organ reserve and complex systemic dysfunction, which has been associated with increased mortality (27).

Both SOFA and LODS scores, widely used for organ dysfunction assessment in critical care, were significantly associated with mortality in our model. SOFA evaluates six organ systems, while LODS focuses on the severity of multiple organ failure. Elevated SOFA and LODS scores reflect deteriorating organ function, a key determinant of ICU mortality. CHF–T2DM patients, prone to metabolic derangement and chronic inflammation, are particularly vulnerable to MODS (28, 29). Our findings reaffirm that incorporating SOFA and LODS into AI models improves early risk stratification and prognostic accuracy (30).

Elevated WBC count was another significant risk factor. This may reflect infection, SIRS, or general physiologic stress. CHF–T2DM patients are often immunocompromised and highly susceptible to infections in the ICU environment (31, 32). WBC elevation may indicate not only infection but also inflammatory dysregulation. It’s important to interpret WBC levels alongside other inflammatory markers, especially given potential “pseudo-normal” values in immunosuppressed or bone marrow-suppressed patients. Early detection and prompt management of infection are essential to reduce mortality in this cohort.

Interestingly, both BMI and creatinine demonstrated negative associations with mortality, suggesting potential protective effects (BMI: *r* = −0.006, *p* = 0.003; creatinine: *r* = −0.024, *p* = 0.020). These findings align with the “obesity paradox” and “reverse epidemiology” frequently reported in critically ill and chronic disease populations (33). Low BMI often reflects malnutrition, inadequate energy reserves, and heightened catabolic stress—all factors associated with worse outcomes (34). In contrast, moderately elevated BMI may enhance survival by providing metabolic reserve and buffering against stress-induced catabolism (33, 35). In our ICU cohort, the overall BMI was lower than in general inpatient or community populations, indicating high disease burden and poor nutritional status (36, 37). Similar protective trends have also been observed in T2DM, where underweight patients face increased insulin resistance and immune impairment (38).

Creatinine, typically a marker of renal dysfunction, paradoxically showed an inverse association with mortality in our model. In the ICU, low creatinine may indicate sarcopenia, chronic wasting, or malnutrition rather than preserved renal function. Given that creatinine is a byproduct of muscle metabolism, its low levels often reflect decreased muscle mass, which correlates with frailty and higher mortality (39). In older adult(s) or chronically ill patients, low baseline creatinine may even obscure the severity of acute kidney injury, further complicating prognostication (40). Thus, both BMI and creatinine serve as indirect indicators of physiological reserve and systemic resilience in critical illness.

Regarding model performance, XGBoost outperformed traditional methods in terms of AUC, accuracy, sensitivity, and specificity. The SHAP analysis offered transparent explanations, highlighting that mild liver disease, LODS, BMI, WBC count, and SOFA were the most influential contributors to the model’s predictions. The SHAP interaction plots further revealed complex interdependencies among variables, providing valuable insights for personalized clinical interventions (41).

This study has several limitations. First, it was a retrospective analysis, which introduces the potential for selection and information bias. Second, the data were derived from three hospitals in Henan Province, which may limit generalizability. Third, the dynamic evolution of clinical variables and treatment responses were not captured. Future work should explore time-series modeling and the integration of intervention data to enhance predictive precision. Finally, although the model demonstrates strong predictive performance and potential clinical utility, it is important to note that improved patient outcomes driven by model-guided care have not yet been established. Future prospective randomized clinical trials are required to validate its real-world clinical impact. Regarding model calibration, the observed slope (1.3832) and intercept (0.3575) indicate that the model’s predicted probabilities were slightly under-dispersed and marginally underestimated the absolute risk. This is likely attributable to the relatively limited sample size of our validation cohort and the inherent complexity of ICU mortality baseline risks. Nevertheless, the robust net benefit observed in the Decision Curve Analysis (DCA) from 15 to 65% thresholds confirms that the model’s discriminatory power translates effectively into actionable clinical value, allowing clinicians to optimize interventions better than conventional default strategies.

## Conclusion

In this study, we developed and validated an explainable machine learning model using the XGBoost algorithm to predict in-hospital mortality among ICU patients with coexisting congestive heart failure (CHF) and type 2 diabetes mellitus (T2DM). The model demonstrated superior predictive performance compared to traditional algorithms, with SHAP (SHapley Additive exPlanations) analysis identifying seven clinically relevant predictors: BMI, myocardial infarction, mild liver disease, SOFA score, LODS score, white blood cell count, and serum creatinine. Notably, our findings confirmed the prognostic significance of organ dysfunction, inflammation, and myocardial injury, while also revealing the protective associations of higher BMI and creatinine, reflecting the obesity paradox and the impact of sarcopenia. The integration of SHAP values enhanced model transparency, making the predictions more interpretable and clinically applicable. These results highlight the potential of explainable machine learning to support early risk stratification and personalized decision-making in critically ill patients with complex comorbidities. Further external validation and real-world implementation are warranted to confirm the model’s generalizability and utility.

## Data Availability

The raw data supporting the conclusions of this article will be made available by the authors, without undue reservation.

## Supplementary material

Supplementary Figure S1 Calibration plot.

Supplementary Figure S2 DCA plot.
