# Supplementary material for: A machine learning–driven and explainable prediction model for in-hospital mortality in patients with diabetes mellitus and congestive heart failure: integrating XGBoost and SHAP approaches
Source: Front Public Health. 2026 Oct 7;14:1951635. doi: 10.3389/fpubh.2026.1951635 (PMC13683621; doi:10.3389/fpubh.2026.1951635)
Supplement: Supplementary file 1 [file Supplementary_file_1.docx]

**Supplementary Table S1 Hyperparameter search spaces, final optimal hyperparameters, and software versions for all evaluated machine learning models**

| ****Algorithm**** | ****Software Package & Version**** | ****Hyperparameter Search Space**** | ****Final Optimal Value**** |
| --- | --- | --- | --- |
| XGBoost | xgboost (v 1.7.3) | learning_rate: [0.01, 0.05, 0.1, 0.2]  max_depth: [3, 5, 7, 9]  n_estimators: [50, 100, 200, 300]  subsample: [0.6, 0.8, 1.0] | learning_rate: 0.05  max_depth: 5  n_estimators: 100  subsample: 0.8 |
| KNN | scikit-learn (v 1.2.2) | n_neighbors: [3, 5, 7, 9, 11]  weights: ['uniform', 'distance']  p (metric): [1 (Manhattan), 2 (Euclidean)] | n_neighbors: 5  weights: 'distance'  p: 2 |
| SVM | scikit-learn (v 1.2.2) | C (Regularization): [0.1, 1, 10, 100]  kernel: ['linear', 'rbf']  gamma: ['scale', 'auto', 0.1, 0.01]  probability: [True] | C: 1.0  kernel: 'rbf'  gamma: 'scale'  probability: True |
| CatBoost | catboost (v 1.1.1) | iterations: [100, 200, 500]  learning_rate: [0.01, 0.05, 0.1]  depth: [4, 6, 8, 10] | iterations: 200  learning_rate: 0.05  depth: 6 |
| AdaBoost | scikit-learn (v 1.2.2) | n_estimators: [50, 100, 200]  learning_rate: [0.01, 0.1, 1.0, 2.0] | n_estimators: 100  learning_rate: 0.1 |

**Supplementary Figure S1 Calibration Plot**

**Supplementary Figure S2 DCA Plot**
